# The effects of climate and land cover on hazel dormouse (*Muscardinus avellanarius*) body mass over space and time

**DOI:** 10.1038/s41598-026-43706-2

**Published:** 2026-03-12

**Authors:** Elizabeth R. Gillie, Danielle Smith, Lisa Worledge, Ian White, Nida Al-Fulaij, Emily Marnham, Orly Razgour

**Affiliations:** 1 Biosciences, Hatherly Laboratories, University of Exeter, Prince of Wales Road, EX4 4PS Exeter, UK; 2 Bat Conservation Trust, Studio 15 Cloisters House, 8 Battersea Park Road, SW8 4BG London, UK; 3 People’s Trust for Endangered Species, 3 Cloisters House, 8 Battersea Park Road, SW8 4BG Battersea, London, UK

**Keywords:** Body size, Climate change, Landscape management, Monitoring, Muscardinus avellanarius, Climate sciences, Ecology, Ecology, Environmental sciences

## Abstract

Climate and land-use change are driving global biodiversity declines, impacting species’ distributions, behaviours, and physiology. The hazel dormouse (*Muscardinus avellanarius*) is highly sensitive to environmental change, yet little is known about how climate change and land-use change influence its body mass. Using data from a 30-year citizen science monitoring programme, we investigated spatiotemporal trends in dormouse body mass across Britain and assessed the roles of climate and land cover as potential drivers. Furthermore, we tested whether changes in body mass vary between sexes and across seasons due to differences in energetic requirements. We show that post-hibernation body mass (May-June) has declined over time, in line with the general observed trend of body size decreasing with rising ambient temperatures to support thermoregulation. In contrast, pre-hibernation body mass has increased over time, potentially indicating increased resource availability linked to increased summer rainfall. Post-hibernation body mass was higher in warmer and wetter sites, while pre-hibernation body mass was higher at cooler and wetter sites. Landscape analysis showed a positive correlation between dormice body mass and the presence of hedgerows 4–6 m tall, while there was a negative effect of overgrown hedgerows. This study provides the first insights into how climate and landscape affect hazel dormouse body mass, offering crucial guidance for conservation strategies amidst ongoing environmental change. Differences in response to climate change across the year highlight the importance of accounting for seasonal variability when studying impacts of climate change on heterothermic animals.

**Supplementary Information:**

The online version contains supplementary material available at 10.1038/s41598-026-43706-2.

## Introduction

Climate change and land-use change are key threats to global biodiversity. Both factors exert pressures on individuals, populations and habitats, often resulting in changes to species’ distributions^1^, phenology^2^and morphology (Gardner et al., 2011^3,58^. Among these responses, changes in body mass have received increasing attention, as body mass reflects individual condition, energetic state and ecological interactions and can respond rapidly to environmental variation^3^. Body mass is a fundamental biological trait that influences key life-history characteristics, including fertility, lifespan and survival, particularly in small mammals where energetic constraints are strong^4^. Unlike structural body size, body mass can vary substantially within individuals over short time periods in response to food availability, climatic conditions and energetic demands. As a result, changes in body mass can provide early indicators of environmental stress and population-level responses to environmental change (Gardner et al., 2011^4^. Understanding how and why species respond to environmental changes through shifts in body mass is essential for predicting ecological and evolutionary outcomes under current and future global change scenarios.

Climatic variables, such as ambient temperature and precipitation, can influence body mass both directly, through thermoregulatory costs, and indirectly by altering resource availability, phenology and foraging opportunities (Gardner et al., 2011). For instance, warmer ambient temperatures and altered rainfall regimes can influence the abundance and quality of food resources through production and plant and invertebrate phenology^5^, thereby affecting body mass, growth and individual condition. Many animals respond to high ambient temperatures through behavioural thermoregulation, whereby they reduce activity or move to cooler areas. These behavioural strategies can be costly as they reduce foraging times and thus reduce their energy intake. The effects of climate on body mass may vary across seasons, particularly in species with strong seasonal cycles in activity, reproduction and energy storage.

Seasonality is especially important for heterothermic mammals, whose energetic demands and physiological constraints differ markedly across the annual cycle^8^. For hibernating species, body mass largely reflects the accumulation of fat reserves during the growing season, which must sustain individuals through winter hibernation and influence condition upon emergence in spring. Climatic conditions experienced during summer can affect the ability to accumulate sufficient reserves, while winter conditions determine the rate at which stored energy is depleted^10^. Therefore, climate during both summer and winter can influence body mass at critical life-stages, with potential consequences for survival and reproductive success^11^. Despite its importance, relatively few studies have examined how climatic conditions shape body mass before and after hibernation, or how these relationships may change over time with ongoing climate change.

Habitat characteristics, including the quality and quantity of available resources, also play a critical role in determining the body mass of individuals. Animals in degraded or resource-scarce habitats, such as agricultural landscapes or urban areas, may experience stunted growth or reduced body mass due to nutritional constraints^12^. Conversely, structurally diverse landscapes that include a mix of woodland, shrub cover and linear features such as hedgerows can support sequential seasonal food availability and movement^13^, particularly for semi-arboreal or arboreal small mammals^14^. Changes in body mass can affect survival, reproduction and resource competition, ultimately shaping community structure and ecosystem function^12,15^. Therefore, understanding these trends is crucial not only for species conservation but also for broader ecosystem management.

Here, we explore temporal and spatial trends in hazel dormouse (*Muscardinus avellanarius*) body mass across Britain. Hazel dormice are small, nocturnal rodents that occur at low population densities throughout much of Europe and northern Turkey and are considered fully arboreal when they are active^16^. They lack a caecum, which inhibits their ability to digest cellulose, and as a result their diet predominantly comprises of seeds, fruit, nuts and invertebrates (Goodwin et al., 2020) ^55^. Hazel dormice hibernate over winter in nests on the ground and between 40 and 70% of the population can be lost during hibernation (Juskaitis 1999) ^57^. Reproduction typically begins in late spring, with first litters of four young occasionally recorded in May and June but are predominantly recorded in August and September. Juveniles will start to disperse in October prior to hibernation. In Britain, hazel dormouse populations have declined by 70% since 2000^17,18^. Their range has shrunk substantially and they are now largely restricted to Southern England and Wales, though some are also found further north in the Midlands and Lake District. Despite these alarming trends, little is known about how their body mass may be changing due to anthropogenic climate and land-use change.

Specifically, we aim to determine if changes in hazel dormouse body mass have occurred over time and space, assess whether these patterns differ between key seasonal phases of the annual cycle, and identify the potential drivers of these trends. We hypothesised that dormouse body mass is affected by climate and land-use changes. However, we also hypothesised that changes in body mass vary between sexes and across seasons due to differences in energetic requirements associated with reproduction and hibernation. We predicted that dormouse body mass will decrease with increasing ambient temperatures, either over space or over time due to climate change, to support thermoregulation. We also predicted that dormouse body mass will increase with availability of preferred habitat types, including woodland, shrub cover, and hedgerows, due to higher resource availability. By explicitly accounting for the seasonal context, our study provides a more nuanced understanding of how climate and land-use change influence body mass in a hibernating mammal.

## Methods

### Dormouse body mass data

To investigate changes in hazel dormouse body mass over time and space, we used pre-existing data collected through the National Dormouse Monitoring Programme (NDMP), a citizen science programme run by the People’s Trust for Endangered Species (PTES). No new data were collected for this study. NDMP monitoring sites (*n*= 706) are distributed across England and Wales, with most sites located in broadleaf and mixed woodlands. Sites have been monitored for 1–36 years from 1988 to 2023. We excluded sites surveyed during the first five years of the programme (1988–1992) to avoid confounding year effects with site effects caused by a limited number of survey locations during these earlier years (number of sites < 30). For our temporal analysis, we included data from 1993 to 2023, covering the full sampling period. For our spatial analysis, we only included data from 2010 to 2023 to avoid biases due to the effect of time and the increase in ambient temperatures and changes in land use in Britain over the past few decades. We followed the approach in Paltrinieri et al.^19^, only including in the temporal analysis, sites with a minimum of five years of records and at least five records per year to ensure sufficient sample size for the statistical analysis and representation of variation. For the spatial analysis we kept sites with a minimum of five records over the spatial analysis time frame of 2010 onwards. Our temporal analysis looks at trends over time and then links them to changes in climate and land-use, while the spatial analysis looks in more detail at drivers across space to understand the effect of ambient temperature, precipitation and land cover on dormouse body mass.

The NDMP follows standard sampling protocols. Dormouse nest boxes are set up in grids of on average between 50 and 100 nest boxes per site and are checked up to once a month from May to October by licensed volunteers, with at least one pre-breeding survey in May/June and one post-breeding survey in September/October. Data are collected on the number of dormice observed, along with biometric data such as sex, age class and body mass. Age class is split into juvenile for > 28 days old, prior to first hibernation season, and adult for individuals post-hibernation > 8–12 months old. More detailed monitoring methods can be found in the NDMP guidelines (PTES, 2024). This dataset represents a subset of the dormouse population, focusing only on individuals using nest boxes within the monitored woodlands. While these data may not capture trends across all habitats, they provide robust insights into woodland populations.

We restricted our analysis to records of adult dormice, excluding those recorded as juveniles. Due to issues in the dataset where some juveniles have been incorrectly recorded as adults in earlier years, we also used a 15 g weight cut off to exclude any unrecorded juveniles^20^. Additionally, we only retained records where sex was recorded and omitted females identified as pregnant to avoid potential bias from pregnancy-related weight variation, although it is difficult to identify pregnant females until they are close to giving birth, so there will be pregnant females within the dataset. Body mass was log-transformed for all analyses.

To account for distinct environmental influences and variation in body mass in the hibernating dormouse, we created two seasonal groups to investigate seasonal body mass differences over time and space. The first group was a pre-hibernation season, October-November, when dormice begin building fat reserves in preparation for hibernation. The second group was a post-hibernation season, representing stable body-mass in late spring to early summer, May–June.

## Climate and land-use data for the temporal analysis

For the climate-driven temporal analysis focusing on understanding the effects of climate change over time, we extracted monthly air temperature and precipitation data from the CHELSA 2.1 monthly dataset^21^. From these, we calculated mean air temperatures and total precipitation corresponding to the summer and winter periods preceding the pre-hibernation and post-hibernation seasons: mean summer air temperatures to represent the active season from the data for July, August and September to relate to pre-hibernation body mass, and winter air temperatures during peak hibernation for December, January and February to relate to post-hibernation body mass. Neither variable showed a clear trend of change over the 31 years sampling period (Supplementary Materials Fig. S1). We also downloaded monthly gridded snow-cover data from the Met Office HadUK-Grid dataset^44^ . The snow variable represents the monthly count of days with greater than 50% of the ground covered by snow at 09:00 UTC. For each study site, values were extracted for December, January and February and summed to derive a total snow-covered days metric during peak hibernation.

For the land-use change temporal analysis, we extracted land cover change data from the UK Centre for Ecology and Hydrology (UKCEH) Land cover change 1990–2015 maps at a 25 m resolution (https://www.ceh.ac.uk/services/land-cover-change-1990-2015). We quantified temporal change (from 1990 to 2015) in the percentage urban and forest cover surrounding each site within a 500 m radius.

## Climate and environmental data for the spatial analysis

For the spatial analysis, carried over the entire study area and the time period of 2010–2023, we extracted bioclimatic data for each site as long term climatological means for the time-period 1981–2010 from the CHELSA 2.1 climatology dataset^21^. We selected four bioclimatic variables to correspond with the seasons of interest: mean daily mean near-surface air temperature of the warmest quarter (BIO10), mean daily mean near-surface air temperature of the coldest quarter (BIO11), mean monthly precipitation of the warmest quarter (BIO18) and mean monthly precipitation of the coldest quarter (BIO19). These variables are provided ready-made in the climatology dataset, varying spatially but not temporally.

We considered a range of habitat structure variables which we obtained from the UKCEH Land Cover map at a 25 m resolution (2021; *Land Cover Map 2021 (25 m rasterised land parcels*,* GB) - EIDC*). As our spatial analysis was restricted to 2010–2023, we only used contemporary land cover variables in this analysis. Of the 21 broad habitat classes, we chose deciduous woodland, coniferous woodland, improved grassland, acid grassland, calcareous grassland, fen, heather, heather grassland, arable and urban. We used the National Forest Inventory (NFI; *National Forest Inventory GB 2020 | Forestry Commission*) for additional forest types, including coppice, shrubs, young trees and felled trees. We calculated hedgerow metrics from the UKCEH’s Land Cover Plus: Hedgerows dataset^22^. This dataset contains information on the length of hedgerows of bands of different height classes from 0.5 m to > 6 m tall. We used the length of each height class as seven additional variables and calculated total hedgerow length across all height classes (see Table S1 in Supplementary Materials).

We calculated the percentage land cover of all habitat variables (from UKCEH and NFI) and hedgerow lengths within three buffer sizes around each site: 500 m, 1 km and 2 km. We chose these buffers to encompass how dormice might interact with their environment at different spatial scales, because understanding a species’ habitat needs requires examining both fine-scale details and broader landscape patterns. Dormice can travel over 300 m when foraging in one night, so we chose 500 m as a finer-scale buffer, then 1 km and 2 km to reflect changes at the meta-population-level in the wider landscape. We calculated two additional landscape metrics using the *‘landscapemetrics’* package in R^23^, mean patch size and aggregation index, for each of the key habitat variables: broadleaf woodland, acid grassland, fen, heather, heather grassland, coppice, shrubs, young trees and felled trees. We further calculated the Simpson’s index across all forest types using the NFI dataset, as our measure of forest habitat diversity; for clarity, we refer to this metric as ‘habitat diversity’ throughout.

### Data analysis

We constructed different models to investigate variation in body mass over time (temporal analysis), to identify climatic and land cover change variables that affect body mass changes over time (climate- and land cover-driven temporal analyses), and then to assess which climatic and land cover variables affect body mass over space. We used Linear Mixed Effects models (LMMs) with the ‘*lme4*’ R package to analyse log-transformed body mass in all analyses. Site was included as a random intercept in all temporal and spatial LMMs to account for repeated measures and non-independence of samples. Year and its interaction with Site were included as additional random intercepts in spatial LMMs to account for residual temporal variability. The response variable in all models was body mass, which was log-transformed to improve normality. Model fits were compared using Akaike Information Criterion (AIC) and stepwise model deletions were performed to select the best model. Prior to analysis, all continuous explanatory variables were standardised by subtracting the mean and dividing by the standard deviation.

We validated LMMs following standard procedures checking for normally distributed residuals, homoscedasticity and any leverage using Cook’s distance. We calculated variance inflation factors using the ‘*performance*’ R package^25^. All simple effect sizes and their standard errors are reported on the log scale.

## Temporal analysis

To investigate long-term trends in dormouse body mass in each season, we fitted weighted LMMs using the ‘nlme’ R package^24^. We specified a variance structure to model homoscedasticity by using a weighted model to account for different residual variances for each level of season (two levels: pre hibernation Oct-Nov and post-hibernation May-Jun). The fixed structure of the model included Year, Season and Sex, as well as all two- and three-way interactions among these variables, to investigate temporal trends, within-year variation (pre- versus post-hibernation) and potential sex-specific patterns in body mass change (sexual dimorphism). We specified a variance structure in LMMs to model heteroscedasticity by allowing different residual variances for the two seasonal levels (Oct-Nov and May-Jun).

To investigate whether the observed changes in body mass over time are linked to climate and land-cover trends, we ran two separate sets of analyses for each season (post- and pre-hibernation). These analyses tested whether ambient temperature, precipitation, snow-covered days and/or land-cover change can explain temporal trends. Firstly, for the climate-driven temporal analysis, we tested the effects of the relevant scaled mean monthly climate variables: winter climate (mean air temperature and precipitation) and total number of snow-covered days for post-hibernation body mass, and summer climate for pre-hibernation body mass. These climate-only LMMs included all two- and three-way interactions between the two climatic variables and Sex. The analysis with total snow-covered days was run in a separate model because it correlated with mean air temperature. We compared the AIC of the climate-only model against another model with year as an additional fixed effect to determine whether time or climate better explained variation in body mass trends. Secondly, for the land-cover-driven analysis, we similarly created models with the temporal change in urban and forest cover, running a separate LMM for each season. We again used AIC to compare the performance of the land-cover change model against the year model. This comparative approach allowed us to assess whether specific environmental drivers provided a superior explanation for the long-term changes in body mass compared to year only.

### Spatial analysis

We then ran a spatial analysis to investigate which climatic and environmental variables affected body mass over space. Although air temperature and precipitation were considered in the temporal analysis to assess its role in long-term trends, the spatial analysis addresses geographic variation across sites during the recent period. We split our analysis into two parts. Firstly, we investigated the impact of climate on body mass separately for each season to capture distinct environmental influences during the post-hibernation and pre-hibernation periods. In the post-hibernation analysis, we used the air temperature of the coldest quarter (BIO11) and precipitation of the coldest quarter (BIO19) as predictors and included the three-way interaction between them and Sex. In the pre-hibernation analysis, we used air temperature of the warmest quarter (BIO10) and the two-way interaction with Sex. We could not include the precipitation of the warmest quarter (BIO18) within the same model because it was collinear with temperature (correlation > 0.7), so created another model with the precipitation x Sex interaction and compared the model fit between them.

Secondly, we investigated the effect of habitat and land cover on body mass across three spatial scales. We restricted this part of the analysis to dormouse records in the post-hibernation season of more constant body mass within the year (May-June). At each scale, habitat variables were checked for collinearity, and any pair with a correlation > 0.7 were excluded, and the more relevant variable to dormouse ecology was retained (Tables S2, S3 and S4 in Supplemental Material). We ran LMMs with the final candidate variables and included Site and Year as random effects. We implemented a stepwise model selection process using the ‘*MASS*’ R package whereby we ranked the combinations of the 18 variables using AIC to identify the best performing set of land cover variables.

## Results

### Temporal analysis

The temporal analysis included 165 sites with more than five years of data and more than five records per year. The dataset included 10,607 and 13,157 body mass records for females and males, respectively. The mean body mass of hazel dormice was 20.4 g (range 15–41.9 g) for females and 20.4 g (range 15.1–44.0 g) for males.

**Fig. 1:**
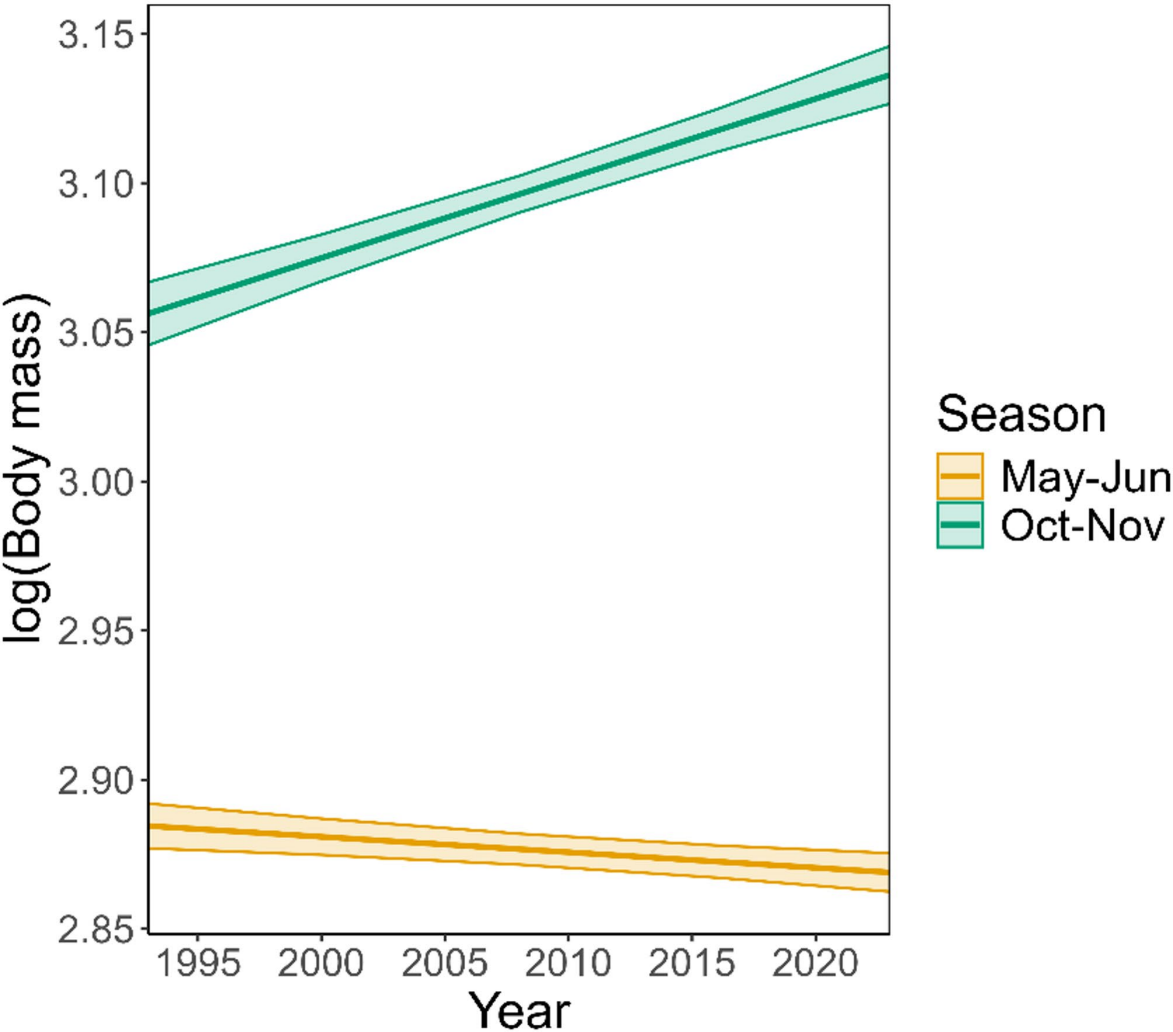
Temporal trends in adult dormouse log body mass per season in study sites across Britain between 1992 and 2023. Straight lines show the fitted trends from the LMM for each season with confidence intervals.

The temporal analysis revealed a significant two-way interaction between year and season on adult dormouse body mass (LMM: *F*_1,23592_ = 131.2, *p* < 0.001, Fig. 1). There was no difference between the sexes (LMM: *F*_1,23592_ = 0.60, *p* = 0.45). For both males and females, post-hibernation body mass (May-June) decreased over time (Effect size (log scale) = −0.003 ± 0.0004; equalling 1 g decrease) and pre-hibernation (October-November) body mass increased over time (Effect size (log scape) = 0.003 ± 0.0003; equalling 1 g increase).

### Climate-driven temporal analysis

Post-hibernation body mass had no evidence of temporal changes associated with winter air temperature or precipitation. In the full three-way interaction model, neither mean winter air temperature nor mean winter precipitation had significant main effects on body mass (air temperature: *F*_1,8264.9_ = 1.87, *p* = 0.17; precipitation: *F*_1,5899.0_ = 0.83, *p* = 0.36). The number of snow-covered days also had no effect on post-hibernation body mass (*F*_1,9552_ = 1.58, *p* = 0.21). The best model only had sex as a significant predictor, with males being heavier than females by 1.03 g (*F*_1,10440.3_ = 55.67, *p* < 0.001). The model including year provided a better fit to the data than models containing the climate variables (AIC_year = −20,173.1 vs. AIC_climate = −20,168.5). Thus, ambient temperature, precipitation and the number of snow-covered days during hibernation did not explain interannual variation in post-hibernation body mass.

**Fig. 2:**
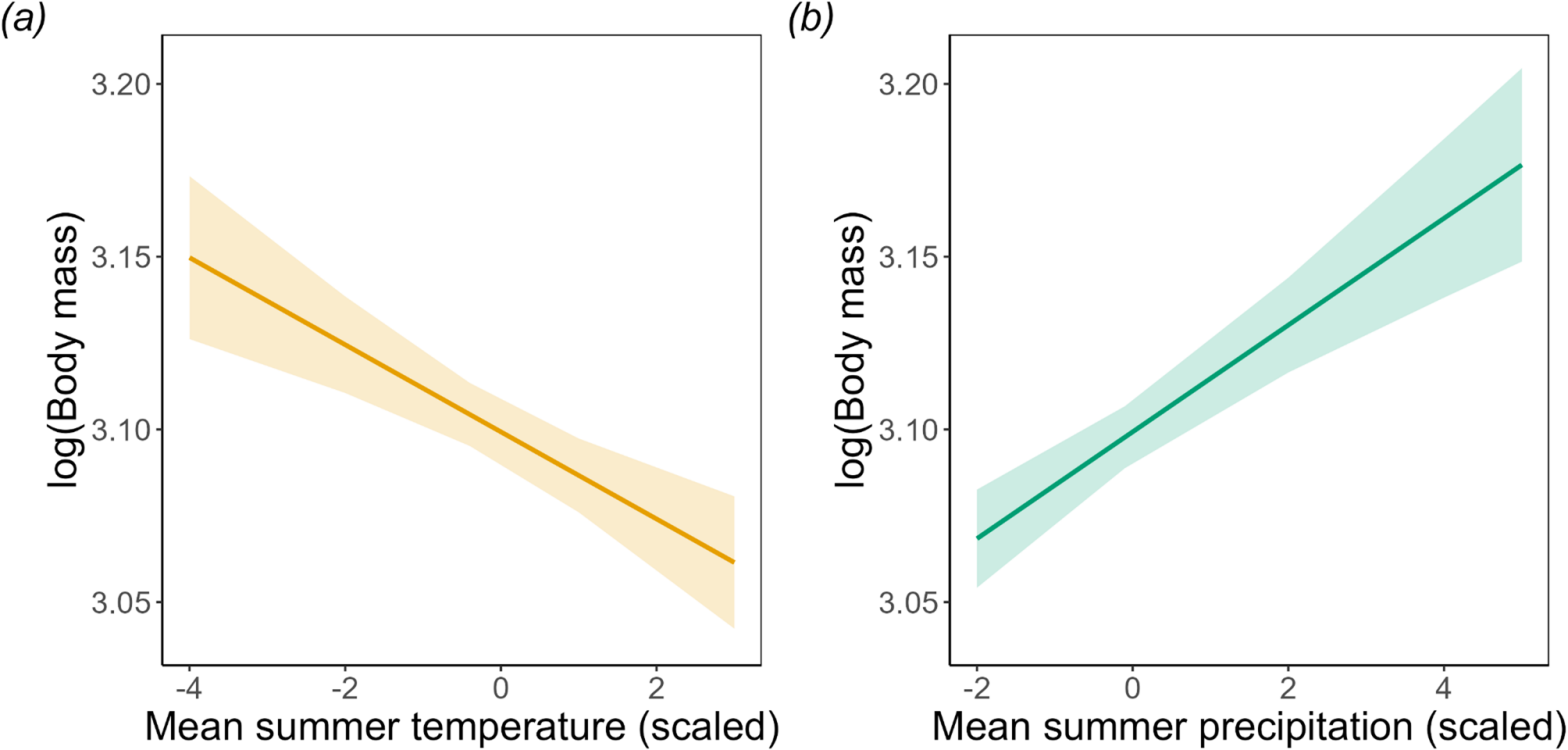
Trends in adult dormouse body mass during pre-hibernation (October-November) with mean summer temperature (**a**) and mean summer precipitation (**b**). Error bars represent ± 1 standard error.

In contrast, pre-hibernation body mass had clear associations with mean summer air temperature and precipitation over time. Higher summer air temperatures were associated with lower pre-hibernation mass (Effect size = −0.013 ± 0.003, *F* = 19.93, *p* < 0.001; Fig. 2A), whereas higher summer precipitation was associated with higher mass (Effect size = 0.015 ± 0.003, *F* = 31.86, *p* < 0.001; Fig. 2B). Model comparison indicated that the climate-driven model performed better than a model including year alone (AIC_climate = −3046.4 vs. AIC_year_only = −3009.3). Including both climate variables and year improved model fit further (AIC = −3088.5), suggesting that climate variables captured additional variance in pre-hibernation body mass beyond temporal trends alone.

### Land cover-driven temporal analysis

Changes in surrounding land cover (urban or forest cover change) did not explain temporal variation in post-hibernation body mass (Forest cover change: *p* = 0.13; urban cover change: *p* = 0.18). No model including land-cover change variables improved model fit relative to simpler models and models with year. Similarly, pre-hibernation body mass had no detectable association with land cover change. Neither changes in urban cover (*p* = 0.72) nor forest cover (*p* = 0.14) were significant predictors and their inclusion did not improve model performance.

### Spatial analysis

#### Climate

**Fig. 3:**
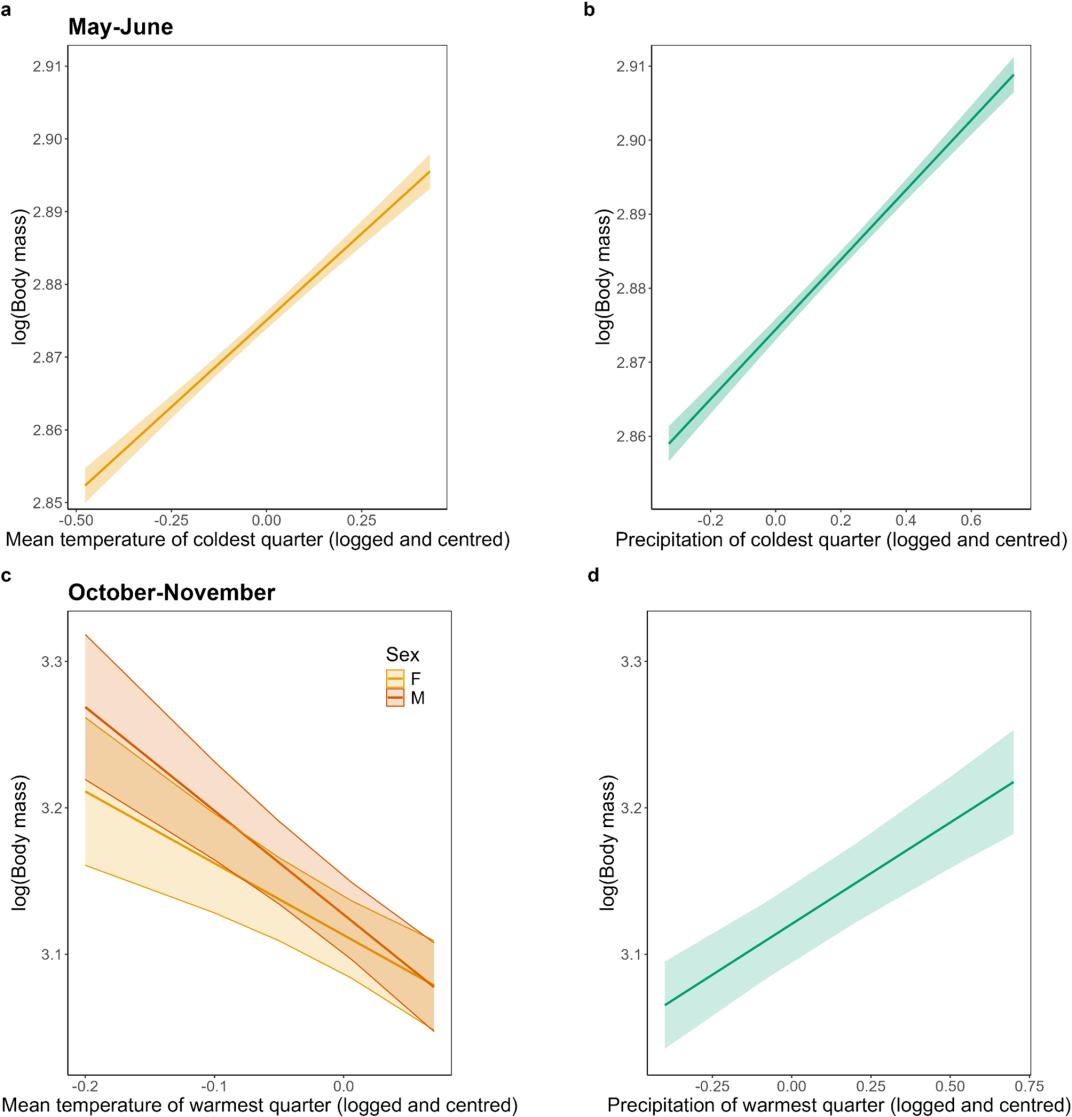
Trends in adult dormouse body mass (logged) over space during post-hibernation (May-June, a and b) and pre-hibernation (October-November, c and d) with temperature and precipitation of the coldest quarter and temperature and precipitation of the warmest quarter, respectively. Predictor variables (mean temperature of warmest and coldest quarters and precipitation of coldest quarter) were logged and mean-centred. Error bars represent ± 1 standard error.

In the analysis of the impact of ambient temperature and precipitation on dormouse body mass across space for the post-hibernation period, we found that air temperature of the coldest quarter had a positive effect on body mass (LMM: Effect size = 0.048 ± 0.02, *F*_1,374_ = 8.22, *p* = 0.004; Fig. 3A). Precipitation had a similar effect on dormouse body mass, whereby body mass increased by 0.047 g for every unit increase in precipitation during the coldest quarter (LMM: *F*_1,356_ = 22.03, *p* < 0.001; Fig. 3B). Air temperatures over the warmest quarter had a contrasting effect on body mass during the pre-hibernation period. Warmer air temperatures led to decreased body mass, and this effect was larger for males (Effect size: −0.71 ± 0.10) than females (Effect size: −0.49 ± 0.10) (LMM: *F*_1,9425_ = 4.47, *p* = 0.03; Fig. 3C). Mean precipitation of the warmest quarter also had an effect on body mass but could not be included due to collinearity. The model with precipitation (AIC = −3844) had better fit than the model with ambient temperature (AIC = −3828). Precipitation had an opposite effect to summer air temperatures, whereby pre-hibernation body mass was higher at sites with higher rainfall over the warmest quarter (Effect size = 0.13 ± 0.002, *F* = 65.47, *p* < 0.001; Fig. 3D).

### Land cover

**Fig. 4:**
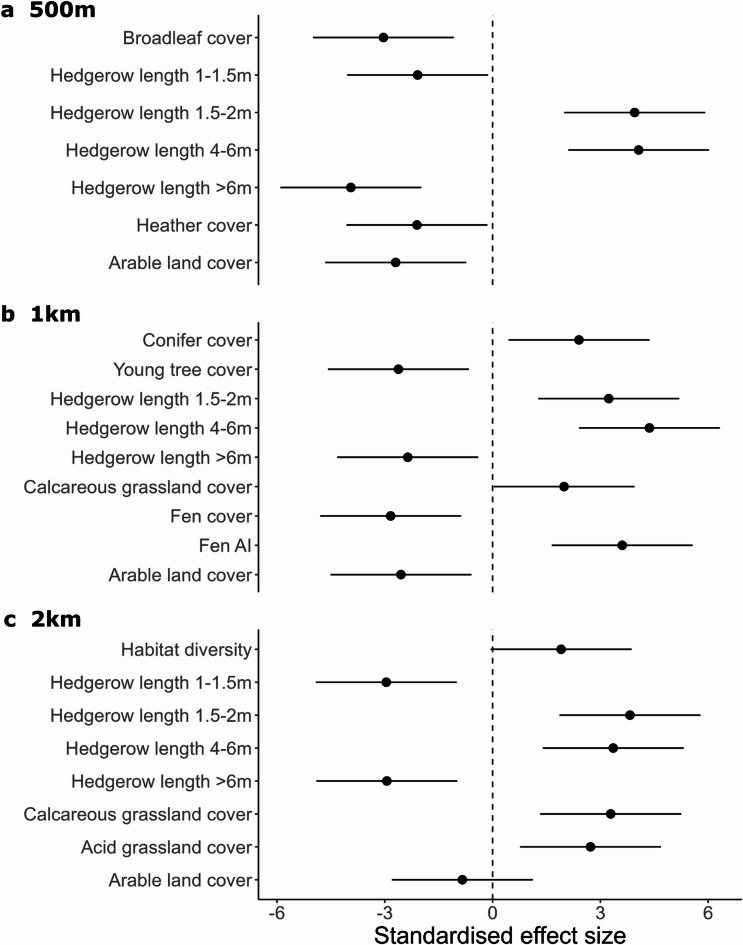
Standardised parameter coefficients from the best linear mixed model used to assess the environmental drivers of adult dormouse body mass in Britain at 500 m (a), 1 km (b) and 2 km (c) scales. Variables are ordered in ecological order from more natural woody habitats to grasslands and modified land (but some variables differ slightly between models). Error bars display 95% confidence intervals around coefficients. Those that overlap the dashed line (marked at zero) indicate non-significant effects, where *p* > 0.05. The figure does not include effect size for categorical significant variables (median hedgerow type).

**Fig. 5:**
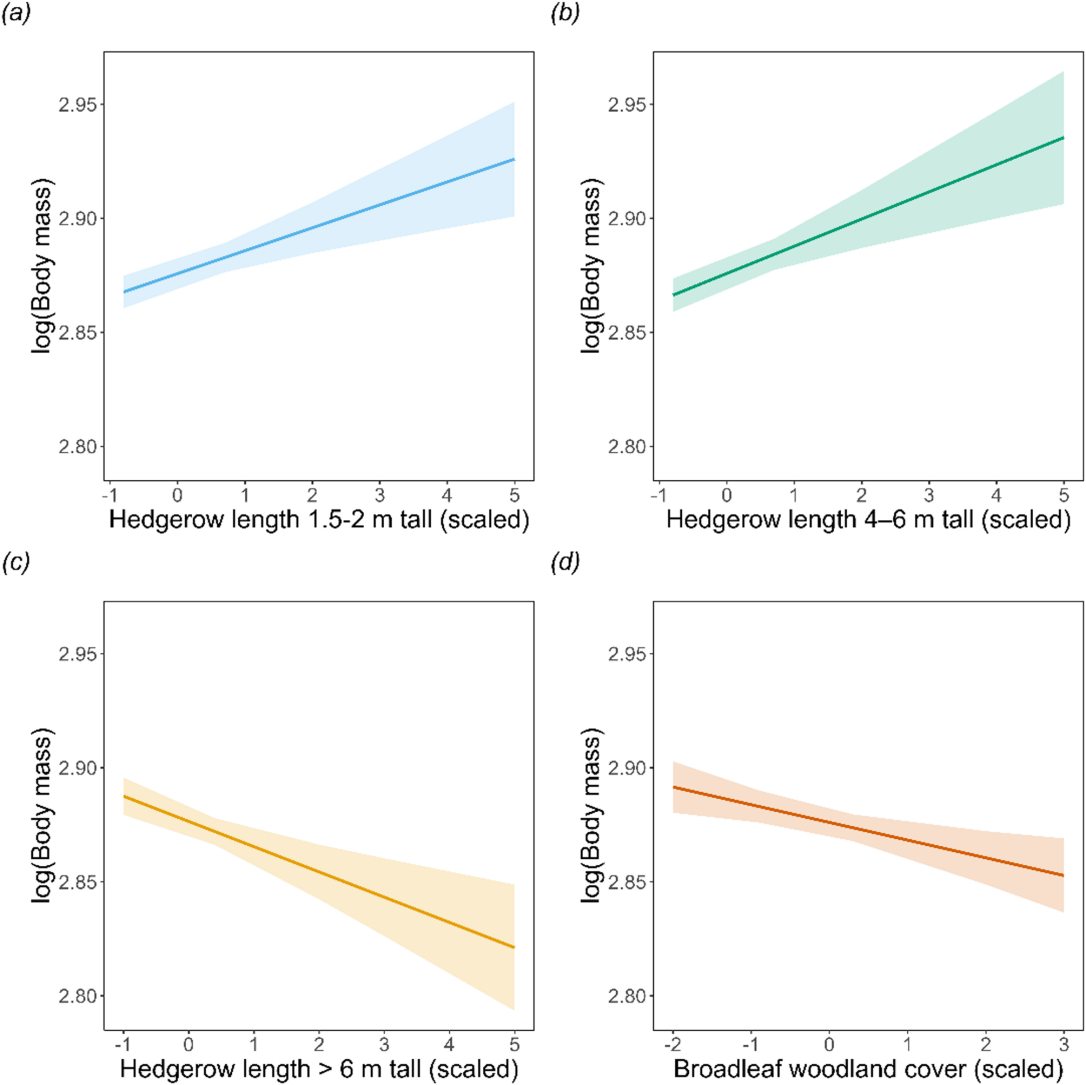
Trend in adult dormouse body mass (logged) with length of hedgerows between 1.5 m and 2 m tall (**a**), 4–6 m tall (**b**), over 6 m tall (**c**) and broadleaf woodland cover (**d**) within a 500 m buffer around sites. Error bars represent ± 1 standard error.

Dormouse body mass was higher in areas with higher density of hedgerows between 1.5 m and 6 m across all spatial scales (Figs. 4 and 5A-C; Table 1), and with higher conifer cover at the 1 km scale. Dormouse body mass was also higher in areas with higher fen aggregation index (1 km scale), calcareous grassland at both 1 km and 2 km scales, and with higher density of acid grassland at the 2 km scale (Fig. 4C). Additionally, habitat diversity had a positive effect at the 2 km scale (Fig. 4C).

**Table 1:** Summary of Linear Mixed Model (LMM) results for effects of land cover variables on dormouse body mass. Variables are ordered in ecological order from more natural woody habitats to grasslands and modified land.

Spatial scale	Habitat variable	Effect size ± SE	DF	F-value	*p*-value
500 m	Broadleaf woodland cover	−0.008 ± 0.003	1,332.51	9.23	0.003
	Hedgerow 1–1.5 m	−0.007 ± 0.003	1,362.97	4.35	0.037
	Hedgerow 1.5–2 m	0.010 ± 0.003	1,375.91	15.63	< 0.001
	Hedgerow 4–6 m	0.012 ± 0.003	1,308.23	16.52	< 0.001
	Hedgerow > 6 m	−0.011 ± 0.003	1,351.48	15.56	< 0.001
	Heather cover	−0.005 ± 0.003	1,256.20	4.43	0.036
	Arable land cover	−0.007 ± 0.002	1,322.85	7.27	0.007
1 km	Conifer cover	0.007 ± 0.003	1,267.06	5.78	0.017
	Young tree cover	−0.006 ± 0.002	1,266.93	6.87	0.009
	Hedgerow 1.5–2 m	0.010 ± 0.003	1,321.37	10.45	0.001
	Hedgerow 4–6 m	0.014 ± 0.003	1,293.52	19.05	< 0.001
	Hedgerow > 6 m	−0.009 ± 0.004	1,289.99	5.57	0.012
	Calcareous grassland cover	0.004 ± 0.002	1,314.23	3.95	0.048
	Fen cover	−0.006 ± 0.002	1,521.06	8.05	0.005
	Fen aggregation index	0.010 ± 0.003	1,273.02	13.01	< 0.001
	Arable land cover	−0.007 ± 0.002	1,292.19	6.50	0.011
2 km	Habitat diversity	0.005 ± 0.002	1,311.82	3.82	0.052
	Hedgerow 1–1.5 m	−0.012 ± 0.004	1,246.13	10.36	0.001
	Hedgerow 1.5–2 m	0.014 ± 0.004	1,331.72	21.67	< 0.001
	Hedgerow 4–6 m	0.012 ± 0.004	1,320.91	8.6	0.004
	Hedgerow > 6 m	−0.012 ± 0.004	1,310.71	8.63	0.004
	Acid grassland density	0.006 ± 0.002	1,365.64	10.49	0.001
	Calcareous grassland cover	0.008 ± 0.002	1,348.37	8.64	0.003
	Arable land cover	−0.002 ± 0.003	1,306.30	3.67	0.056

In contrast, dormouse body mass was lower in areas with higher density of hedgerows over 6 m tall (all scales) and hedgerows between 1 and 1.5 m at 2 km scale, and higher broadleaf woodland cover at 500 m. Dormouse body was also lower in areas with higher arable land cover at all spatial scales (Fig. 4B and C), and young tree cover and fen cover at the 1 km scale (Fig. 4B).

## Discussion

Using body mass data from a long-term monitoring programme of hazel dormice populations across England and Wales, we found that the average body mass of hazel dormice decreased over 31 years, but only during the post-hibernation period (May-June). During the pre-hibernation season (Oct-Nov), dormouse body mass has increased since 1992. Changes in climatic variables only explained part of these patterns, while land cover changes did not explain any changes in body mass. Across space, our analysis revealed that dormice are heavier in areas with warmer and wetter climates during the post-hibernation season and in areas with higher summer rainfall and colder summer air temperatures during the pre-hibernation season. Only the latter pattern differs between sexes, with body mass of pre-hibernation males showing a steeper decline with increasing summer air temperatures than females. Additionally, we found that dormouse body mass is lower in areas with higher density of hedgerows over 6 m tall within the surrounding landscape of dormice sites, and higher in areas with higher density of hedgerows between 1.5 m and 6 m tall. Interestingly, broadleaf woodland cover has a negative effect on body mass at a fine scale, though dormice body mass is higher at sites with higher habitat diversity at the broader landscape, within 2 km around the site. Our findings suggest a complex interplay between climate and land cover in determining trends in dormouse body condition, offering new insights into how these changes are driven by climatic and habitat variables.

### Effects of climate change and land cover change over time

Our analysis of temporal trends in dormouse body mass revealed contrasting seasonal patterns over time. We identified long-term declines in dormice body mass during the post-hibernation period from May-June. Britain has warmed by on average 1 °C over the last ~ 30 years^27^, suggesting a link between dormouse body mass and increasing ambient temperatures. However, neither air temperature nor rainfall during the winter hibernation period explained inter-annual variation in post-hibernation body mass. Although winter snow cover can reduce energy expenditure during torpor by improving insultation, interannual variation in snow conditions across the study sites was low, and winter snow cover did not explain body mass either. The identified seasonal decline in body mass differs from findings of previous studies on other rodents that found body mass generally increased over time^28^. A review of studies of birds and mammals found that the body size of 72% of the birds and 65% of the mammals studied decreases with increasing air temperatures, suggesting discrepancies across taxa. Although we found no effect of average winter air temperatures on dormouse body mass, the observed decline in body mass over the 30 years period could be a result of warmer and more erratic winter air temperatures resulting in animals rousing more frequently during hibernation, which uses up more fat reserves^5,30^. Warmer, wetter winters may also increase rainfall rather than snowfall, potentially soaking hibernation nests and increasing heat loss. This results in dormice emerging from hibernation in poorer condition^5^.

The observed body mass declines in spring may also be the result of earlier flowering of plants in Britain^32^, leading to phenological mismatches between dormouse activity and food availability in the post hibernation period. Dormice are considered specialist feeders and what they eat depends on what is seasonally available and abundant at the time. In spring, dormice typically depend on flowers and pollen from hawthorn and then move on to oak, honeysuckle and sycamore^33^. A potential early summer shortage of insects like caterpillars and aphids, which dormice depend on before berries become available, may also exacerbate this trend.

In contrast, pre-hibernation body mass (October-November) has increased over time, which could be linked to improved resource availability in late summer if either climate change or changes in habitat management results in greater resources locally. Warmer summers may extend the availability of key food sources such as berries and nuts^34^. However, we found that dormouse body mass decreased with increasing summer air temperatures, suggesting that summer rainfall has a stronger impact on resource availability for dormice, despite its effect on reducing dormice activity length by 3 min/mm^35^. Rainfall affects the production of hazelnuts from hazel trees, which require sufficient amounts of rainfall during summer to avoid water stress and the associated decreased kernel size, yields and nut quality^36,37^. Alternatively, ambient temperature can influence energy balance in opposing ways during the active season. Moderate warming may reduce thermoregulatory costs, whereas extremely high absolute ambient temperatures can constrain energy intake by limiting heat dissipation and reducing foraging efficiency^38^. We did not see a considerable change in mean summer ambient temperatures over time, but further work would benefit from investigating the effect of heat waves and extreme temperatures on hazel dormice in the UK. The observed pre-hibernation increase in body mass over time suggests that in heterothermic animals, climate change could impact ontogenetic development differently across seasonal periods, creating contrasting outcomes for body condition.

Changes in dormouse body mass over time can also be a response to changes in land cover and habitat structure, affecting habitat quality and resource availability. Although most of the study sites are located in broadleaf and mixed woodlands, the woodlands likely experienced changes in management practices over the years. In particular, cessation of coppicing practices, woodland maturation and conversion to conifer affect resource availability for dormice at different times of the year^16^. On the other hand, improved woodland and site management practices may contribute to better resource quality and availability, ultimately enhancing body condition during the critical pre-hibernation period. However, at the wider landscape scale, we found no effect of changes in forest and urban cover on dormouse body mass.

The observed change in dormouse body mass during pre-hibernation and post-hibernation periods could have implications for this species. The decline in post-hibernation body mass over the past 30 years could negatively impact dormouse survival and reproductive success. Given that reproductive success in dormice is likely to be influenced by post-hibernation body mass as reproduction takes place directly afterwards, the decline in post-hibernation body mass identified in our study may explain the observed declines of dormice numbers in Britain^18^. Previous studies have found a positive association between body mass and survival in bats^39^, which suggests our observed decline in body mass could have fitness implications. Declining body mass could result in decreased survival and depressed population growth rates^40^. Decreases in adult body condition could thus impact the productivity and function of ecosystems^4^.

The effects of climate change on hazel dormice extend beyond shifts in body mass and seasonal resource availability. Future projections suggest that climate change will alter the distribution of dormice in the UK, with changes in climate impacting their range^41^. While future climate conditions could enable dormice to establish in new areas, reductions in body mass may affect dispersal ability, limiting their capacity to colonise these newly suitable regions^42^. Smaller-bodied individuals may have reduced energy reserves and lower endurance, making long-distance dispersal more challenging^43^, particularly in fragmented landscapes where dormice already face barriers to movement.

### Effects of climate over space

We show that both ambient temperature and rainfall affect trends in body mass of adult hazel dormouse across space. In the post-hibernation season (May-June), dormice are heavier in sites experiencing higher winter air temperatures and winter rainfall, while in the pre-hibernation season (Oct-Nov) dormice are heavier in sites experiencing higher summer rainfall and lower summer air temperatures. The positive relationship we found between winter air temperatures and rainfall and dormouse post-hibernation body mass over space contradicts the trend we found of decreasing post-hibernation body mass over time, given the increase in winter rainfall over the past few decades^27^. Moreover, findings from our spatial analysis contradict Combe et al.,’s (2023) finding that warmer and wetter winters negatively affect dormouse survival and population growth rates over time. Hence, there appears to be discordance between spatial and temporal patterns of changes in dormouse post-hibernation body mass.

In contrast, the relationship between pre-hibernation body size and climatic conditions over space mirrors what we found in our temporal analysis, highlighting the strong effect of summer air temperatures and rainfall on dormice body mass. Drier conditions during the summer affect resource availability for dormice. Low rainfall can reduce vegetation productivity, especially in the production of hazelnuts or cobnuts from hazel trees, by causing water stress in the trees, leading to decreased kernel size, lowers yields and poorer quality nuts due to impaired physiological processes in the plant^36,37^. Additionally, drier conditions may increase thermoregulatory stress, leading to higher metabolic costs and greater expenditure of energy, which could contribute to lower body mass^24^. This in turn could affect survival rates during hibernation. On the other hand, the frequency of short torpor increases with colder and wetter summer conditions, which can reduce the number of breeding events and juvenile counts, but is not related to adult body mass. Steeper decline in male pre-hibernation body mass with increasing summer air temperatures across space indicates that males are more strongly affected by changes in resource availability during the summer with increasing air temperatures. Males have larger home ranges than females, are more mobile and share their home ranges with a larger number of other individuals^45^, hence likely to experience stronger competition for resources.

### Effects of land cover over space

Hedgerow management emerged as a key predictor of dormouse body mass, highlighting the importance of habitat and landscape features for both local-scale foraging and broader-scale dispersal. Across all spatial scales, dormice body mass is higher in areas with higher availability of hedgerows between 1.5 m and 6 m tall, and lower in areas with hedgerows over 6 m tall. Taller hedgerows often indicate a lack of management, leading to gappy structures dominated by overgrown and mature trees. This results in the loss of low, shrubby cover essential for dormouse shelter, food and movement corridors^46^. Undermanaged hedgerows have already been identified as a threat to British wildlife, with only 50% of hedgerows in England assessed as being in good conditions in 2007 (RSPB^47^^48^;. Well-managed hedgerows provide critical connectivity between habitat patches and support the sequential seasonal food availability that dormice require throughout the year^46^. The negative correlation with smaller hedgerows (< 1.5 m) implies that the over-management of hedgerows could also be a threat. Indeed, studies show that hedgerow cutting regimes shifted to every three years helped maintain the number of flowers and biomass of berries of hawthorn hedges^49^. However, whilst hedgerow management came out as a key predictor, there have been no in-depth studies of dormice living in hedgerows, and therefore it is unknown what may be driving the relationship. Although well-managed hedgerows are likely better for dormice, they likely benefit the dormouse populations living within or in close proximity to them, rather than a few hundred meters away. Well-managed, species rich hedgerows may enable dormouse populations to be resident in hedges rather than just being able to use them for dispersal. More evidence and monitoring are required to fully understand the importance of hedgerows in the landscape mosaic near dormouse sites.

Somewhat surprisingly, adult dormouse body mass is negatively related to broadleaf woodland cover at a fine spatial scale. Increasing broadleaf woodland cover may be associated with lower body mass for several reasons. Firstly, as broadleaf woodlands mature, their canopy closes, reducing understory vegetation^50^. This leads to a decline in key food sources, such as flowers and fruits, which dormice rely on^51^. Dormice require woodlands and hedgerows with high tree and shrub diversity to provide continuous food resources throughout the year. With the cessation of coppicing practices across much of Britain, this vegetation diversity, and consequently food diversity, has been reduced^52^. Secondly, our measure of land cover may not capture the complexity of woodland habitats as it only classifies them to the broadleaf category and not to species level. The positive relationship between dormouse body mass and woodland diversity at the broader scale suggests that habitat diversity is more important than overall woodland cover, at least for dispersal. High habitat diversity can provide the sequential seasonal food availability that dormice need throughout the year^33^.

### Benefits and pitfalls of longitudinal citizen science data

Citizen science data, such as that used in this study, can provide invaluable insights into long-term trends in species responses to environmental change^53^. However, there are inherent biases in volunteer-collected data that must be considered. One key challenge is the difficulty in identifying pregnant females in the field, as pregnancy is typically only apparent when individuals are heavily gravid. This limitation may introduce biases in the dataset. However, unlike previous studies of bats^19^, in this study, we did not detect differences between males and females in their response to climate both over space and time, suggesting that the potential inclusion of heavier early-pregnancy females did not bias our results. This highlights the importance of also studying males to identify broader population trends without the confounding effects of reproductive status. Additionally, the accuracy of age classification in monitoring programmes, like the National Dormouse Monitoring Programme, can vary, particularly in earlier years of programme establishment when juveniles may be misclassified as adults by less experienced surveyors. Despite these limitations, our analyses showed no differences in trends when individuals under 15 g were excluded, suggesting that misclassification had minimal impact on the overall results. Despite their limitations, long-term monitoring datasets remain an essential tool for tracking the responses of species to climate change. These datasets provide critical, large-scale information that would be difficult to obtain through short-term studies alone, allowing researchers to detect patterns and changes over time that can inform conservation strategies and policy decisions.

## Conclusions

Our findings provide a comprehensive insight into how climate and land cover influence hazel dormouse body mass across temporal and spatial scales. Differences in response to climate between the post-hibernation and pre-hibernation season highlight the importance of accounting for seasonal variability when assessing impacts of climate change on heterothermic animals. Our spatial analyses demonstrate the critical roles of ambient temperature, precipitation, and landscape variables in determining adult dormouse body mass. Our results stress the need for fine-scale habitat management and conservation strategies to ensure the survival of this sensitive species in a rapidly changing environment. This work highlights the crucial ongoing role that species monitoring programmes can play in tracking, recording and mitigating anthropogenic stressors on biodiversity.

## Supplementary Information

Below is the link to the electronic supplementary material.

Supplementary Material 1

## Data Availability

The data associated with this paper and code to replicate the analysis is deposited in Figshare: https://doi.org/10.6084/m9.figshare.29899949.
