# Supplementary material for: The effects of climate and land cover on hazel dormouse (Muscardinus avellanarius) body mass over space and time
Source: Sci Rep. 2026 Mar 12;16:9800. doi: 10.1038/s41598-026-43706-2 (PMC13018304; doi:10.1038/s41598-026-43706-2)
Supplement: Supplementary file 1 — Supplementary Material 1 [file 41598_2026_43706_MOESM1_ESM.pdf]

Supplementary Materials for '**The effects of climate and land cover on hazel dormouse (*Muscardinus avellanarius*) body size over space and time'**

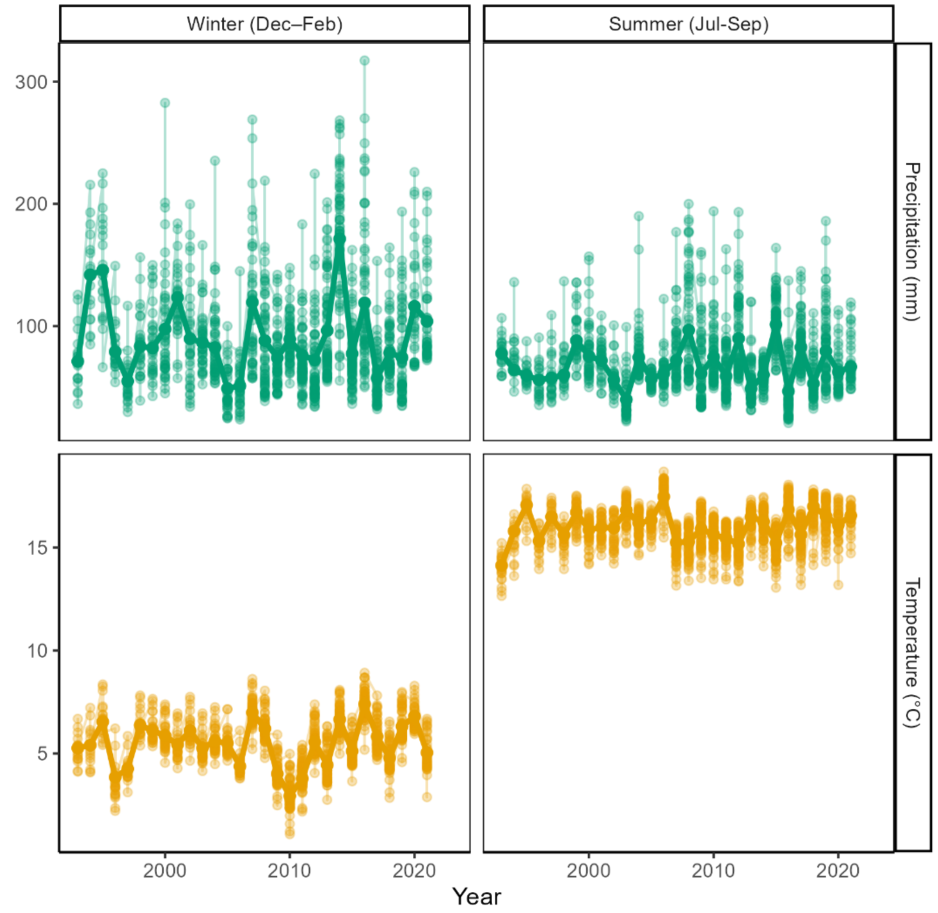

Figure S1. Trends in mean monthly temperature and precipitation in winter (December-February) and summer (July-September) across all sites used in temporal analysis. These periods represent climate likely to impact dormouse body mass during hibernation and active periods, respectively.

Table S1. List of all variables used in analysis and their sources.

Variable	Source	Description
Deciduous Woodland	UKCEH Land Cover Map 2021[a]	Broad habitat class; % land cover within 500m, 1km, 2km buffers
Coniferous Woodland	UKCEH Land Cover Map 2021[a]	Same as above
Improved Grassland	UKCEH Land Cover Map 2021[a]	Same as above
Acid Grassland	UKCEH Land Cover Map 2021[a]	Same as above
Calcareous Grassland	UKCEH Land Cover Map 2021[a]	Same as above
Fen	UKCEH Land Cover Map 2021[a]	Same as above
Heather	UKCEH Land Cover Map 2021[a]	Same as above
Heather Grassland	UKCEH Land Cover Map 2021[a]	Same as above
Arable	UKCEH Land Cover Map 2021[a]	Same as above
Urban	UKCEH Land Cover Map 2021[a]	Same as above
Coppice	National Forest Inventory (NFI) 2020[b]	Forest type; % land cover within buffers
Shrubs	NFI 2020[b]	Same as above
Young Trees	NFI 2020[b]	Same as above
Felled Trees	NFI 2020[b]	Same as above
Hedgerow Length (0.5–1m)	UKCEH Land Cover Plus: Hedgerows (1)	Length of hedgerows in this height class within buffers
Hedgerow Length (1–2m)	Same as above	Same as above
Hedgerow Length (2–3m)	Same as above	Same as above
Hedgerow Length (3–4m)	Same as above	Same as above
Hedgerow Length (4–5m)	Same as above	Same as above
Hedgerow Length (5–6m)	Same as above	Same as above

Hedgerow Length (>6m)	Same as above	Same as above
Total Hedgerow Length	Same as above	Sum of all height classes
Mean Patch Size	Calculated using 'landscapemetrics' in R	For selected habitats (e.g., broadleaf woodland, fen, etc.)
Aggregation Index	Calculated using 'landscapemetrics' in R	For same selected habitats
Simpson's Diversity Index	Calculated from NFI data	Measures habitat diversity across forest types

[a]2021; Land Cover Map 2021 (25m rasterised land parcels, GB) - EIDC

[b]National Forest Inventory GB 2020 | Forestry Commission

Table S2. Correlations between spatial variables 2km. Top variables with R > 0.7. Those marked in bold were removed from the analysis. NFI_5 and NFI_6 were cut as not enough sites with these habitat types

Variable 1	Variable 2	R value
**NFI_9**	CEH_2	0.762819
CEH_1	**CEH_mean_patch_size_1**	0.733046
CEH_6	**CEH_mean_patch_size_6**	0.773348
CEH_7	**CEH_mean_patch_size_7**	0.809884
CEH_9	**CEH_mean_patch_size_9**	0.886244
CEH_8	**CEH_mean_patch_size_8**	0.859613
CEH_aggregation_index_8	**CEH_mean_patch_size_8**	0.809997
CEH_5	**CEH_mean_patch_size_5**	0.873335
CEH_10	**CEH_mean_patch_size_10**	0.94975
**NFI_mean_patch_size_5**	**NFI_5**	0.708679
**NFI_mean_patch_size_6**	**NFI_6**	0.99999
**NFI_mean_patch_size_7**	NFI_7	0.882416
**NFI_aggregation_index_7**	NFI_7	0.718973
**NFI_aggregation_index_7**	**NFI_mean_patch_size_7**	0.738349
HLength_1c	**Total_Hedgerow_Length**	0.786622
HLength_2	**Total_Hedgerow_Length**	0.77378
HLength_4	**Total_Hedgerow_Length**	0.820664
HLength_6	**Total_Hedgerow_Length**	0.811846
HLength_1c	**HLength_1a**	0.779027
HLength_3	**HLength_2**	0.705038
HLength_4	**HLength_2**	0.719837

Table S3. Correlations between spatial variables 500m. Top variables with R > 0.7. Those marked in bold were removed from the analysis. NFI_5 and NFI_6 were cut as not enough sites with these habitat types.

Variable 1	Variable 2	R value
**CEH_mean_patch_size_5**	CEH_5	0.985767
**CEH_mean_patch_size_6**	CEH_6	0.818066
**CEH_mean_patch_size_7**	CEH_7	0.930772
**CEH_aggregation_index_7**	CEH_7	0.724172
**CEH_mean_patch_size_8**	CEH_8	0.956627
**CEH_mean_patch_size_9**	CEH_9	0.936554
**CEH_mean_patch_size_10**	CEH_10	0.911108
**CEH_mean_patch_size_8**	CEH_aggregation_index_8	0.726009
**NFI_5**	**NFI_mean_patch_size_5**	0.962571
NFI_aggregation_index_5	**NFI_mean_patch_size_5**	0.701093
**NFI_6**	**NFI_mean_patch_size_6**	1
NFI_aggregation_index_6	**NFI_mean_patch_size_6**	1
NFI_7	**NFI_mean_patch_size_7**	0.834686
NFI_aggregation_index_7	**NFI_mean_patch_size_7**	0.736993
NFI_aggregation_index_8	**NFI_mean_patch_size_8**	0.735742
NFI_9	**NFI_mean_patch_size_9**	0.887302
NFI_7	**NFI_aggregation_index_7**	0.723579
NFI_6	**NFI_aggregation_index_6**	1
**Total_Hedgerow_Length**	HLength_1c	0.745615
**Total_Hedgerow_Length**	HLength_2	0.741381
**Total_Hedgerow_Length**	HLength_4	0.764262
**Total_Hedgerow_Length**	HLength_6	0.820913

Table S4. Correlations between spatial variables at 1km scale. Top variables with R > 0.7. Those marked in bold were removed from the analysis. NFI_5 and NFI_6 were cut as not enough sites with these habitat types.

Variable 1	Variable 2	R value
CEH_1	**CEH_mean_patch_size_1**	0.7330463
CEH_2	**NFI_9**	0.7628188
CEH_5	**CEH_mean_patch_size_5**	0.8733354
CEH_6	**CEH_mean_patch_size_6**	0.7733476
CEH_7	**CEH_mean_patch_size_7**	0.8098843
CEH_8	**CEH_mean_patch_size_8**	0.8596125
CEH_9	**CEH_mean_patch_size_9**	0.8862438
CEH_10	**CEH_mean_patch_size_10**	0.9497502
**CEH_mean_patch_size_8**	CEH_aggregation_index_8	0.809997
**NFI_5**	**NFI_mean_patch_size_5**	0.7086787
**NFI_6**	**NFI_mean_patch_size_6**	0.9999899
**NFI_7**	**NFI_mean_patch_size_7**	0.8824158
**NFI_7**	NFI_aggregation_index_7	0.7189731
**NFI_mean_patch_size_7**	NFI_aggregation_index_7	0.7383488
**Total_Hedgerow_Length**	HLength_1c	0.7866219
**Total_Hedgerow_Length**	HLength_2	0.7737797
**Total_Hedgerow_Length**	HLength_4	0.820664
HLength_1a	**HLength_1c**	0.7790266
HLength_2	HLength_3	0.7050375
HLength_2	HLength_4	0.7198374

References

1. Broughton RK, Burkmar R, McCracken M, Mitschunas N, Norton LR, Pallett DW, et al. UKCEH Land Cover Plus: Hedgerows 2016-2021 (England). NERC EDS Environmental Information Data Centre.; 2024.
